# Volatile Organic Compounds and Pulmonary Function in the Third National Health and Nutrition Examination Survey, 1988–1994

**DOI:** 10.1289/ehp.9019

**Published:** 2006-04-25

**Authors:** Leslie Elliott, Matthew P. Longnecker, Grace E. Kissling, Stephanie J. London

**Affiliations:** National Institute of Environmental Health Sciences, National Institutes of Health, Department of Health and Human Services, Research Triangle Park, North Carolina, USA

**Keywords:** air fresheners, air pollution (indoor), deodorants, 1,4-dichlorobenzene, exposure, environmental exposure, FEV_1_, lung function, respiratory function tests, VOC

## Abstract

**Background:**

Volatile organic compounds (VOCs) are present in much higher concentrations indoors, where people spend most of their time, than outdoors and may have adverse health effects. VOCs have been associated with respiratory symptoms, but few studies address objective respiratory end points such as pulmonary function. Blood levels of VOCs may be more indicative of personal exposures than are air concentrations; no studies have addressed their relationship with respiratory outcomes.

**Objective:**

We examined whether concentrations of 11 VOCs that were commonly identified in blood from a sample of the U.S. population were associated with pulmonary function.

**Methods:**

We used data from 953 adult participants (20–59 years of age) in the Third National Health and Nutrition Examination Survey (1988–1994) who had VOC blood measures as well as pulmonary function measures. Linear regression models were used to evaluate the relationship between 11 VOCs and measures of pulmonary function.

**Results:**

After adjustment for smoking, only 1,4-dichlorobenzene (1,4-DCB) was associated with reduced pulmonary function. Participants in the highest decile of 1,4-DCB concentration had decrements of −153 mL [95% confidence interval (CI), −297 to −8] in forced expiratory volume in 1 sec and −346 mL/sec (95% CI, −667 to −24) in maximum mid-expiratory flow rate, compared with participants in the lowest decile.

**Conclusions:**

Exposure to 1,4-DCB, a VOC related to the use of air fresheners, toilet bowl deodorants, and mothballs, at levels found in the U.S. general population, may result in reduced pulmonary function. This common exposure may have long-term adverse effects on respiratory health.

Volatile organic compounds (VOCs) are a diverse group of chemicals emitted as gases from a variety of commonly used products. The general population is exposed to VOCs from cleaning and degreasing agents, pesticides, air fresheners, toilet bowl deodorants, furniture, tobacco smoke, and building materials such as pressed wood products, adhesives, carpeting, paints, and varnishes. Although VOCs are also released into the outdoor air through automotive exhaust and industrial emissions, indoor VOC concentrations are much higher (Wallace et al. 1987, 1991).

Because people spend most of their time indoors, health effects related to VOCs in the residential setting are a concern, particularly with respect to respiratory illness (Diez et al. 2000; Farrow et al. 2003; Fiedler et al. 2005; Harving et al. 1991; Koren et al. 1992; Norback et al. 1995; Pappas et al. 2000; Smedje et al. 1997; Venn et al. 2003; Wieslander et al. 1997). Several studies have shown that elevated air concentrations of VOCs are associated with respiratory symptoms (Diez et al. 2000; Norback et al. 1995; Pappas et al. 2000; Rumchev et al. 2005; Smedje et al. 1997; Wieslander et al. 1997). Studies of VOC exposures and measures of pulmonary function have mostly been small and have used short-term measurements of VOC air concentrations in a single location to characterize exposures, which may not reflect the chronic exposures to these compounds (Fiedler et al. 2005; Harving et al. 1991; Norback et al. 1995; Pappas et al. 2000; Wieslander et al. 1997). Blood concentrations may better reflect chronic exposures to VOCs because they integrate exposures from all sources and can be used to estimate internal dose (Ashley and Prah 1997; Ashley et al. 1994; Sexton et al. 2005a).

A variety of VOCs were measured in a subset of participants in the Third National Health and Nutrition Examination Survey (1988–1994) (NHANES III) to determine background exposure levels for adults in the general U.S. population (Ashley et al. 1994). Because there is a paucity of information about chronic VOC exposure and pulmonary function, we examined VOC blood concentrations in relation to pulmonary function using data from NHANES III (Ashley et al. 1994).

## Materials and Methods

### Study population

We used data from NHANES III and its component Priority Toxicant Reference Range Study, designed to assess the levels of common pesticides and VOCs in a representative sample of the U.S. adult population. The studies were conducted from 1988 through 1994. Detailed information about NHANES III and the Priority Toxicant Reference Range Study may be found elsewhere [National Center for Health Statistics (NCHS) 1996, 2000]. Briefly, NHANES III is the seventh in a series of periodic surveys conducted by the NCHS of the Centers for Disease Control and Prevention designed to provide national estimates of the health and nutritional status of noninstitutionalized U.S. civilians ≥ 2 months of age. The Priority Toxicant Reference Range Study included a sample of 1,338 men and women from NHANES III, 20–59 years of age, selected on the basis of age, race, sex, and region of residence. Among these 1,338 participants, 1,018 provided an additional blood sample for measurement of VOCs and completed a questionnaire about exposure to various chemical products.

### Pulmonary function

In NHANES III, spirometry was conducted according to the 1987 American Thoracic Society recommendations (NCHS 2001). The National Institute of Occupational Safety and Health (NIOSH) served as the quality control center for the results. Technicians received formal training and satisfactorily completed an NIOSH-approved course on spirometry.

Our analyses included forced expiratory volume at 1 sec (FEV_1_; milliliters), forced vital capacity (FVC; milliliters), peak expiratory flow rate (PEFR; milliliters per second), and maximum mid-expiratory flow rate (MMEFR; milliliters per second). We adjusted all models for race/ethnicity group (indicator variables for African American, Mexican American, and other), age (continuous), age squared (continuous), standing height (continuous), body mass index (continuous), and sex, to account for differences in pulmonary function based on these characteristics.

We included participants in analyses if they had at least two successful pulmonary function maneuvers and if their results were coded as reliable and reproducible. A reliable maneuver was a maximal exhalation without cough, excessive hesitation, leak, obstructed mouthpiece, variable effort, or early termination (NCHS 1996). Reproducible maneuvers were recorded for FVC and FEV_1_ and were defined as the largest FVC and second largest FVC within 5%, and the largest FEV_1_ and second largest FEV_1_ within 5%. Of 1,018 participants with VOC measures, 953 met our pulmonary function inclusion criteria.

### VOCs

In the Priority Toxicant Reference Range Study, 32 VOCs were measured in blood, using purge and trap gas chromatography/mass spectrometry as previously described (Ashley et al. 1992, 1994). We analyzed the 11 VOCs with median values above the limit of detection [1,1,1-trichloroethane (1,1,1-TCE), 1,4-dichlorobenzene (1,4-DCB), 2-butanone, acetone, benzene, ethylbenzene, *m*,*p*-xylene, *o*-xylene, styrene, tetrachloroethene, toluene]. Of the 953 participants who had a value for at least 1 of 11 VOCs and who had acceptable pulmonary function data, sample sizes varied across VOCs (range, 513–953), because results for some VOCs were not available for all participants (NCHS 2001). Although the reasons for different sample sizes are not given in the NHANES III documentation, it is possible that some blood samples failed to meet acceptability standards or were invalid due to clotting, or that the laboratory experienced problems with instruments or quality control parameters (Sexton et al. 2005a).

### Statistical analyses

We used ordinary least-squares regression models to evaluate the association between each VOC and each pulmonary function outcome. For samples with VOC measures below the limit of detection, a value equal to the detection limit divided by the square root of 2 was assigned (NCHS 2000). We used natural log transformations of VOC concentrations to reduce the influence of their skewed distributions on the regression model estimates. We used Wilcoxon rank-sum tests to compare VOC median values between two groups and Kruskal-Wallis tests to compare values among three or more groups. We performed tests for linear trends across deciles using one-way analysis of variance. We conducted all analyses using SAS (version 9.0; SAS Institute Inc., Cary, NC). Weighting is not recommended for analysis of data from the Priority Toxicant Reference Range Study (NCHS 2000).

For descriptive purposes, we first analyzed VOCs in relation to pulmonary function without adjustment for smoking. However, because smoking and environmental tobacco smoke are sources of VOCs and also affect pulmonary function, we then added terms for smoking status (current, quit within the previous 12 months, quit more than 12 months previously, never), number of cigarettes smoked per day (continuous), years smoked (continuous), and serum cotinine level (continuous). Smoking was a confounder for most VOCs.

We then used a change-in-estimate method to evaluate additional variables as confounders for the VOCs still related to pulmonary function after adjustment for smoking (Greenland 1989). Our cutoff criterion was a 10% change in the VOC β-coefficient in relation to pulmonary function. In this manner, we assessed the following potential confounders: socioeconomic status (education, poverty:income ratio, use of food stamps within the previous 12 months), self-reported doctor diagnosis of emphysema, use of fireplace within the previous 12 months or wood or gas stove for heating or cooking, age of the house (construction year before 1946construction year before 1946–1973–1974 to present), presence of furred pets at home, and occupational exposure. Occupational exposure (yes, no) was indicated by a variable denoting occupations associated with chronic obstructive pulmonary disease (COPD) in this population (Hnizdo et al. 2002). The only factor that met the criterion for confounding was self-reported doctor diagnosis of emphysema, and this was included in the final models. We repeated analyses excluding people with self-reported doctor diagnosis of asthma, and the results were not changed appreciably.

## Results

Characteristics of the study population are shown in Table 1. The mean age was 36.6 years (range, 20–59), 43.1% were female, and 26.3% were current smokers.

Table 2 shows distributions of the 11 VOCs with median values above the limit of detection. As expected, acetone was present in much higher concentrations than other VOCs because it is produced endogenously. Men had significantly higher measured values for most VOCs (Wilcoxon rank-sum tests, *p* < 0.05), except for 1,1,1-TCE, 1,4-DCB, and tetrachloroethene (TCE). As has been reported previously for this population (Churchill et al. 2001), Mexican Americans had lower concentrations of benzene, ethylbenzene, styrene, TCE, and toluene and significantly higher levels of *m*,*p*-xylene than did other ethnic groups. For 1,4-DCB, non-Hispanic whites had the lowest and African Americans the highest concentrations.

In the models unadjusted for smoking, reductions in at least one pulmonary function outcome were statistically significant for 1,4-DCB, benzene, ethylbenzene, styrene, and toluene (data not shown). However, when these models were adjusted for smoking variables, only 1,4-DCB remained statistically significantly associated with reduced pulmonary function. For example, after adjustment for smoking, VOC β-coefficients for FEV_1_ changed from −72 mL (*p* < 0.0001) to −1 mL (*p* = 0.95) for benzene, from −51 mL (*p* = 0.03) to 15 mL (*p* = 0.57) for ethylbenzene, from −61 mL (*p* = 0.01) to 42 mL (*p* = 0.19) for styrene, and from −69 mL (*p* < 0.01) to 16 mL (*p* = 0.60) for toluene, whereas the β-coefficient for 1,4-DCB remained unchanged (−24 mL, *p* = 0.04).

Because only 1,4-DCB maintained its association with pulmonary function in the presence of smoking, further analyses were limited to this VOC. The exposure distribution of 1,4-DCB differed by race/ethnicity group (Kruskal-Wallis test, *p* < 0.0001), with African Americans having the highest exposures (Table 3).

Among all participants, 1,4-DCB was inversely related to all four pulmonary function measures but was statistically significant only for FEV_1_ and MMEFR (Table 4). Power is limited for sex-specific analyses; however, higher 1,4-DCB was related to lower levels of each pulmonary function measure in both men and women. Likewise, 1,4-DCB was inversely associated with all four measures within each of the race groupings, although numbers become unstable. Numbers are further reduced within the six race/sex groups, but 1,4-DCB was inversely related to at least one of the four pulmonary function measures in each of the six subgroups. The results were strongest and statistically significant for non-Hispanic white females (FEV_1_, β = −266, *p* = 0.02) and African-American males (FEV_1_, β = −282, *p* = 0.01), although we did not find significant evidence of effect modification by race/sex combinations (multiple partial *F*-test, *p* > 0.10 for all pulmonary function outcomes).

Higher levels of 1,4-DCB were related to reduced pulmonary function in never-smokers as well as smokers (Table 4). Results for never-smokers were similar when we defined nonsmokers in a more stringent manner as having serum cotinine < 0.62 ng/mL, the 75th percentile among nonsmokers (*n* = 299; data not shown).

To further examine the relationship between 1,4-DCB and pulmonary function, we conducted additional analyses using urinary concentrations of 2,5-dichlorophenol (2,5-DCP), the major metabolite of 1,4-DCB (Hissink et al. 1997). 2,5-DCP was one of 12 pesticide metabolites measured in the urine of NHANES III participants, using capillary gas chromatography and tandem mass spectrometry (Hill et al. 1995b). Although 2,5-DCP measurements were available only on 534 of the 846 subjects included in the analysis of 1,4-DCB, the β-coefficients for both compounds were inversely related to all pulmonary function measures, and the result for FEV_1_ was more statistically precise. For example, the expected change in FEV_1_ with each increase in exposure from the 10th to 90th percentile (3.76 μg/L for 1,4-DCB and 4.67 μg/L for 2,5-DCP) was −96 mL (*p* = 0.03) for 1,4-DCB and −134 mL (*p* = 0.02) for 2,5-DCP.

**Figure 1:**
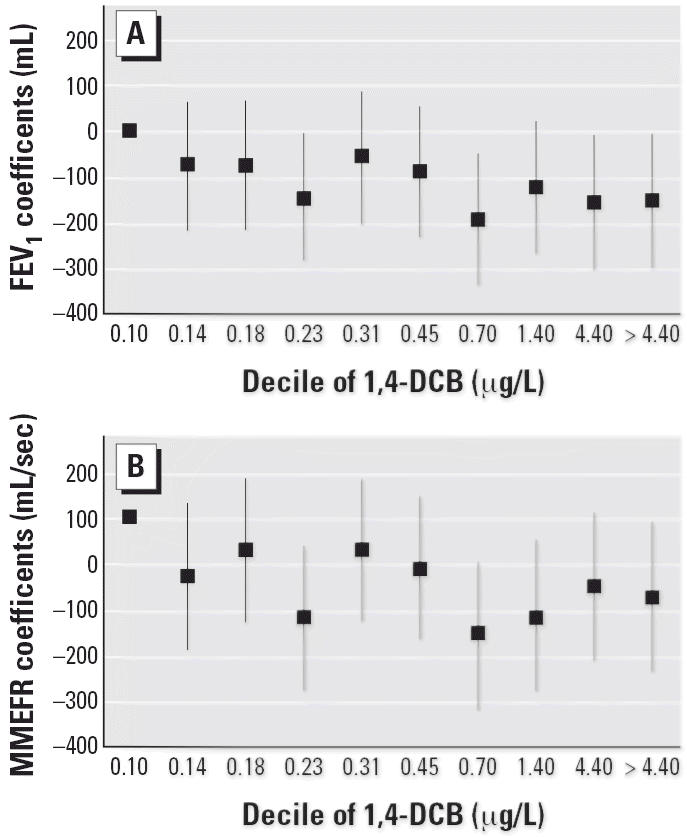
Changes in FEV_1_ (*A*) and MMEFR (*B*) (with 95% CIs) for each decile of 1,4-DCB concentration among 846 participants in the NHANES III (1988–1994).

To facilitate interpretation of the association between 1,4-DCB and pulmonary function that we observed in these data using logarithmic transformation, we categorized nontransformed values of 1,4-DCB into deciles. Figure 1 shows the changes in FEV_1_ (milliliters) and MMEFR (milliliters per second) for each decile of 1,4-DCB exposure, compared with participants in the lowest decile. Tests for linear trend across deciles were statistically significant (FEV_1_, *p* = 0.02; MMEFR, *p* = 0.02). Subjects in the highest decile of exposure had FEV_1_ decrements of −153 mL [95% confidence interval (CI), −297 to −8] and MMEFR decrements of −346 mL/sec (95% CI, −667 to −24), compared with participants in the lowest decile.

## Discussion

We examined the relationship between blood concentrations of 11 VOCs with median values above the limit of detection and pulmonary function outcomes in participants of NHANES III and found that 1,4-DCB was the only VOC associated with reduced pulmonary function after adjustment for smoking. Participants in the highest decile of 1,4-DCB concentration had FEV_1_ and MMEFR decrements of −153 mL (95% CI, −297 to −8) and −346 mL/sec (95% CI, −667 to −24), respectively, compared with participants in the lowest decile. This compares with a 100-mL deficit in FEV_1_ for the highest tertile of serum cotinine in nonsmoking females in the NHANES III population (Eisner 2002).

Because we conducted separate analyses for 11 different VOCs, it is possible that the statistical significance of the inverse association between 1,4-DCB and pulmonary function occurred by chance. However, this seems unlikely given the consistent results across subgroup analyses. Furthermore, an analysis of pulmonary function and 2,5-DCP, the urinary metabolite of 1,4-DCB, resulted in similar associations, with the result for FEV_1_ reaching statistical significance, despite the smaller sample size.

It is possible that 1,4-DCB blood concentrations may reflect exposure better than do blood concentrations of other VOCs, because air and blood concentrations are better correlated for 1,4-DCB (Sexton et al. 2005a). In the School Health Initiative: Environment, Learning, Disease (SHIELD) study, 2-day integrated personal air samples of indoor VOCs were taken immediately before taking VOC blood measurements from 143 children in Minneapolis (Sexton et al. 2005a). Personal air samples and VOC blood measurements were taken four times over 2 years. Among the VOCs measured, only 1,4-DCB had a high correlation between air and blood concentrations (*R*^2^ = 0.79). Except for acetone and 2-butanone, that study measured the same VOCs we included in our analyses.

Although VOCs generally do not persist in the blood after termination of acute exposure (Ashley and Prah 1997), after frequent prolonged exposures, blood concentrations can reflect chronic exposures (Ashley and Prah 1997; Ashley et al. 1994; Sexton et al. 2005a, 2005b). For example, examination of the uptake and elimination of some VOCs has suggested that bioaccumulation may occur in multiple storage sites in the human body (Ashley and Prah 1997). In the SHIELD study, the between-child variability of 1,4-DCB blood concentrations greatly exceeded the within-child variability (ratio = 434), suggesting that one blood measurement of 1,4-DCB is a good indication of an individual’s blood concentration over time. In contrast, other VOCs had much lower ratios of between- and within-child variability; for example, the next highest were for TCE (ratio = 2), ethylbenzene (ratio ~ 1), and 1,1,1-TCE (ratio ~ 1). The high ratio of between- to within-child variability for 1,4-DCB was not seen in the younger children of the Developmental Research on Attention and Memory Skills (DREAMS) study, but far fewer children had more than one blood sample to estimate the ratio of within- to between-child variability (e.g., 126 in the SHIELD study compared with 22 in the DREAMS study) (Sexton et al. 2005b).

Apart from the findings in the SHIELD study (Sexton et al. 2005a), little is known about the relationship between personal 1,4-DCB exposures and blood concentrations. Based on data from a graph of this relationship in children (Sexton et al. 2005a), we estimate that a 1,4-DCB blood concentration of 10 μg/L may correspond to personal exposures of 102 μg/m^3^ or greater, which is close to the proposed chronic duration minimal risk limit (120 μg/m^3^) [Agency for Toxic Substances and Disease Registry (ATSDR) 2004]. Blood levels of 1,4-DCB were higher in children from the SHIELD study than in the NHANES III adults we studied. For example, the 95th percentile was 11.03 μg/L for NHANES III and 27.00 μg/L for the SHIELD study. If 1,4-DCB air concentrations can be extrapolated from blood concentrations, it is possible that the highest blood concentrations of 1,4-DCB in NHANES III represent exposures to air concentrations greater than the proposed chronic duration minimal risk limit.

People who use air fresheners, toilet bowl deodorants, and mothballs have potential for high exposure to 1,4-DCB because it is an important component of these products (ATSDR 2004; Churchill et al. 2001). However, exposure also occurs in the absence of these products as the compound is common in indoor environments. For example, the U.S. Environmental Protection Agency’s Total Exposure Assessment Methodology (TEAM) study in 1987 found 1,4-DCB in the air of 80% of the homes surveyed (Wallace et al. 1987), although only one-third of the homes used products containing 1,4-DCB (Wallace 1991). The finding that 96% of the NHANES III subset had detectable 1,4-DCB blood concentrations (Hill et al. 1995a) is further evidence that exposure is common (Sampson et al. 1994).

Although 1,4-DCB is common in indoor environments, little is known about its effects on human health. Hepatic, dermatologic, and respiratory effects have been reported with acute exposures, but these case reports lack clear information about exposure levels (ATSDR 2004; National Institutes of Health 2005). Data from a single occupational study of 58 men (Hollingsworth et al. 1956) were used in conjunction with animal studies to derive acute and chronic exposure levels in the air considered to pose the minimal risk to humans (ATSDR 2004). Because these limits are derived mostly from animal studies, uncertainty factors are used for extrapolation to humans. The minimal risk limits for human exposure to 1,4-DCB are 2 ppm (12 mg/m^3^) for acute duration (≤ 24 hr), 0.1 ppm (0.6 mg/m^3^) for intermediate duration (> 14 days but < 1 year), and 0.02 ppm (0.12 mg/m^3^) for chronic duration (up to a lifetime) (ATSDR 2004).

Among the studies that have measured 1,4-DCB air or blood concentrations (Delfino et al. 2003; Hollingsworth et al. 1956; Rumchev et al. 2005; Sexton et al. 2005a, 2005b; Wallace et al. 1987, 1991), only three measured health outcomes (Delfino et al. 2003; Hollingsworth et al. 1956; Rumchev et al. 2005). Of these, only two measured respiratory outcomes and included only children. In one study, children 6 months to 3 years of age (*n* = 88) had higher odds of asthma with increasing indoor air concentrations of 1,4-DCB (Rumchev et al. 2005). In a panel study of 22 asthmatic children 10–16 years of age, respiratory symptoms were associated with outdoor air concentrations of total VOCs but not with 1,4-DCB alone (Delfino et al. 2003). Children in this study measured morning and evening peak flow; no relationship with any VOC was observed. No other pulmonary measures were tested. As expected, outdoor air concentrations of 1,4-DCB were low (0.3–3.0 μg/m^3^).

Indoor air concentrations of 1,4-DCB are significantly greater than outdoor air concentrations (Wallace et al. 1987). For example, the TEAM study measured mean personal exposures of 21 μg/m^3^ and indoor concentrations of 30 μg/m^3^, compared with outdoor concentrations of 2.0 μg/m^3^ (Wallace et al. 1987). According to other measures, levels in some homes and public restrooms may reach almost 1.64 mg/m^3^ (ATSDR 2004), which is greater than the minimal risk limit for chronic exposure.

The chronic duration minimal risk limit is based on observed eosinophilic changes in the olfactory epithelium of rats; no information was available on effects of exposure on pulmonary function (ATSDR 2004). Although reductions in pulmonary function can be transient and do not necessarily reflect permanent adverse health effects (ATS 2000), they generally precede permanent effects. Thus, chronic reduction in FEV_1_ is a sentinel event for adverse health effects from inhaled exposures, such as air pollution (ATS 2000). In particular, FEV_1_ has been identified as a risk factor in cardiovascular disease, stroke, and lung cancer, as well as an important predictor of all-cause mortality (Hole et al. 1996).

It is probable that most exposures to 1,4-DCB are chronic, rather than acute and sporadic, because 1,4-DCB is a component of household products used for prolonged periods. For example, air fresheners, toilet bowl deodorants, and mothballs are used until their emissions cease, and then they are replaced. Staff interviewers in the SHIELD study, where blood concentrations of 1,4-DCB were high, noted that many children’s homes had pervasive scents of air fresheners (Sexton et al. 2005a). In NHANES III, 32.1% of the participants in the VOC study reported recent use of air fresheners or room deodorizers. Fewer participants reported recent use of toilet bowl deodorants (8.7%), although their use was associated with a 2-fold increase in odds of having high 1,4-DCB blood levels (Churchill et al. 2001).

Because NHANES III is a cross-sectional study, measurements of exposure and outcome were made at the same time, and it is not possible to determine if 1,4-DCB exposure preceded pulmonary function decline. A longitudinal study measuring pulmonary function and exposure to 1,4-DCB at various time points would be necessary to evaluate the temporality of this relationship. Although it is possible that people who are exposed to toilet bowl or air fresheners and other room deodorizers might also be exposed to cleaning products that impair pulmonary function, we had no data to address this.

The inverse association between 1,4-DCB concentration and pulmonary function may have been affected by unmeasured confounders. We assessed the influence of other factors that may be related to pulmonary function and to 1,4-DCB exposure, such as type of heating, use of wood fires, age of house, presence of furred pets, occupation, socioeconomic status, presence of environmental tobacco smoke, smoking history, and diagnosis of asthma or emphysema. Only emphysema confounded the relationship between 1,4-DCB and pulmonary function deficits. The ability to carefully adjust for smoking with several variables, including the objective measure of environmental tobacco smoke exposure, serum cotinine, was a considerable strength of our analyses.

The size and diversity of this NHANES III sample make it possible to examine the relationships between VOCs and pulmonary function in more detail than has been possible in smaller studies. Our findings suggest that 1,4-DCB exposure at levels found in the U.S. general population may result in decreases in pulmonary function. Larger and longitudinal studies would be necessary to properly evaluate the effects on respiratory symptoms and disease.

## Figures and Tables

**Table 1:** Selected characteristics of participants in NHANES III Priority Toxicant Reference Range Study (1988–1994).

	Males (*n* = 542)	Females (*n* = 411)	Total^a^ (*n* = 953)
Race/ethnicity (%)			
Non-Hispanic white	39.9	38.2	39.1
African American	32.3	31.1	31.8
Mexican American	25.3	26.3	25.7
Other	2.6	4.4	3.4
Smoking status (%)			
Current smokers	29.5	22.1	26.3
Former smokers_1_^b^	1.3	1.2	1.3
Former smokers_2_^c^	15.3	9.3	12.7
Never smokers	53.9	67.4	59.7
Potential confounders (%)			
Diagnosed asthma^d^	7.0	9.0	7.9
Diagnosed emphysema^d^	1.1	0.7	0.9
Presence of furred pets	34.3	35.8	34.9
Occupation with COPD risk	31.5	12.2	22.3
Pulmonary function measures (mean ± SD)			
FEV_1_ (mL)	3,875 ± 732	2,867 ± 544	3,440 ± 825
FVC (mL)	4,858 ± 826	3,516 ± 612	4,279 ± 996
PEFR (mL/sec)	9,374 ± 1,834	6,731 ± 1,292	8,234 ± 2,085
MMEFR (mL/sec)	3,812 ± 1,344	3,023 ± 1,016	3,472 ± 1,275
Age [years (mean ± range)]	36.9 ± 20–59	36.2 ± 20–59	36.6 ± 20–59

a Subjects in this table were included in at least one analysis of VOC blood concentration and pulmonary function.

b Former smokers who quit smoking within the previous 12 months.

c Former smokers who quit smoking > 12 months previously.

d Self-reported doctor’s diagnosis of asthma or emphysema.

**Table 2:** Values of selected VOCs^a^ (μg/L) measured in participants in NHANES III Priority Toxicant Reference Range Study, 1988–1994, limited to participants with pulmonary function data.

		Total		Males				Females			
VOC	LOD	No.^b^	< LOD^c^	No.	Median	10th	90th	No.	Median	10th	90th
1,1,1-TCE	0.09	513	122	292	0.14	0.06	0.54	221	0.13	0.06	0.41
1,4-DCB	0.07	854	38	491	0.33	0.11	3.89	363	0.30	0.10	4.83
2-Butanone	0.50	908	0	515	5.59	2.41	13.72	393	5.36	2.26	11.28
Acetone	200	852	0	479	1,945	801	7,187	373	1,788	769	6,109
Benzene	0.03	743	113	421	0.07	0.02	0.42	322	0.06	0.02	0.26
Ethylbenzene	0.02	570	33	325	0.07	0.03	0.22	245	0.05	0.02	0.16
*m*,*p*-Xylene	0.03	953	362	542	0.13	0.02	0.47	411	0.11	0.02	0.34
*o*-Xylene	0.04	593	24	343	0.11	0.06	0.21	250	0.10	0.06	0.17
Styrene	0.02	589	74	336	0.05	0.01	0.16	253	0.04	0.01	0.10
Tetrachloroethene	0.03	539	133	306	0.07	0.02	0.38	233	0.06	0.02	0.32
Toluene	0.09	540	4	308	0.33	0.14	1.32	232	0.25	0.13	0.88

LOD, limit of detection. 10th and 90th are percentiles.

a Compounds were selected if median values were above the limit of detection. VOCs not meeting inclusion criterion: 1,1,2,2-tetrachloroethane, 1,1,2-TCE, 1,1-dichloroethane, 1,1-dichloroethene, 1,2-DCB, 1,2-dichloroethane, 1,2-dichloropropane, 1,3-DCB, bromodichloromethane, bromoform, carbon tetrachloride, chlorobenzene, chloroform, *cis*-1,2-dichloroethene, dibromochloromethane, dibromomethane, methylene chloride, *trans*-1,2-dichloroethene, and trichloroethene.

b Number of available samples for each VOC. Not all VOCs were measured in every individual, resulting in different sample sizes.

c Number of participants with samples below the limit of detection.

**Table 3:** Distribution of 1,4-DCB by sex and race/ethnicity group in NHANES III Priority Toxicant Reference Range Study, 1988–1994.

	Males					Females				
	No.	Median	Range	10th	90th	No.	Median	Range	10th	90th
Non-Hispanic Whites	200	0.22	0.05–16.98	0.09	1.75	140	0.20	0.05–20.47	0.08	1.66
African Americans	157	0.56	0.09–51.24	0.15	6.56	111	0.52	0.08–46.46	0.16	8.67
Mexican Americans	122	0.34	0.05–51.89	0.10	5.64	98	0.29	0.05–26.52	0.09	7.26

10th and 90th are percentiles.

**Table 4:** Linear regression coefficients [β (95% CI)] for 1,4-DCB^a^ and pulmonary function outcomes in NHANES III, 1988–1994.

	No.	FEV_1_	FVC	PEFR	MMEFR
All participants^b^	846	−96 (−182 to −11)*	−64 (−162 to 33)	−207 (−472 to 58)	−198 (−388 to −8)*
Males	488	−103 (−227 to 21)	−76 (−218 to 65)	−183 (−575 to 209)	−165 (−447 to 117)
Females	358	−82 (−191 to 27)	−46 (−170 to 78)	−211 (−542 to 121)	−238 (−478 to 2)
Whites	334	−155 (−320 to 9)	−112 (−303 to 79)	−181 (−665 to 301)	−320 (−681 to 41)
African Americans	266	−153 (−300 to −6)*	− 99 (−262 to 64)	−517 (−1,016 to −18)*	−307 (−627 to 14)
Mexican Americans	219	−46 (−183 to 90)	−53 (−214 to 108)	65 (−345 to 474)	−58 (−379 to 263)
White males	198	−26 (−260 to 208)	69 (−204 to 343)	−154 (−849 to 540)	−250 (−788 to 288)
White females	137	−266 (−488 to −43)*	−259 (−512 to −7)*	−202 (−855 to 451)	−409 (−879 to 60)
African-American males	156	−282 (−497 to −66)*	−242 (−481 to −3)*	−712 (−1,460 to 35)	−402 (−875 to 70)
African-American females	110	43 (−152 to 239)	113 (−106 to 332)	−242 (−896 to 412)	−154 (−583 to 275)
Mexican-American males	122	−68 (−289 to 152)	−85 (−342 to 172)	214 (−442 to 870)	−48 (−559 to 463)
Mexican-American females	197	−95 (−264 to 74)	−64 (−274 to 146)	−292 (−779 to 196)	−266 (−666 to 134)
Ever smokers	458	−137 (−259 to −16)*	−113 (−250 to 25)	−270 (−647 to 108)	−223 (−495 to 49)
Never smokers	388	−57 (−177 to 64)	−18 (−157 to 122)	−178 (−551 to 195)	−185 (−449 to 79)

a The β-coefficient estimates the expected change in lung function as 1,4-DCB increases from the 10th to 90th percentile (3.76 μg/L) on the natural log scale.

b Includes all race/ethnicity groups. Models were adjusted for race/ethnicity, sex, age, age-squared, standing height, body mass index, self-reported doctor diagnosis of emphysema, smoking status, number of cigarettes smoked per day, years smoked, and serum cotinine levels. Stratified models exclude variables used for stratification.

* β-Coefficient differs from 0 at *p* < 0.05.
